# *In Vitro* Anticancer Activity of Phlorofucofuroeckol A via Upregulation of Activating Transcription Factor 3 against Human Colorectal Cancer Cells

**DOI:** 10.3390/md14040069

**Published:** 2016-03-29

**Authors:** Hyun Ji Eo, Tae-Hyung Kwon, Gwang Hun Park, Hun Min Song, Su-Jin Lee, Nyun-Ho Park, Jin Boo Jeong

**Affiliations:** 1 Department of Bioresource Sciences, Andong National University, Andong 36729, Korea; ehj56@naver.com (H.J.E.); enter0230@hanmail.net (G.H.P.); ou1@dreamwiz.com (H.M.S.); 2 Chuncheon Bioindustry Foundation, Chuncheon 24234, Korea; taehyung0218@naver.com; 3 Department of Herbal Medicine Resource, Kangwon National University, Dogye 25949, Korea; withsj0830@naver.com; 4 Gyeongbuk Institute for Marine Bioindustry, Uljin 36315, Korea; pnh863660@gimb.or.kr

**Keywords:** activating transcription factor 3, apoptosis, human colorectal cancer, phlorofucofuroeckol A, *Eisenia bicyclis*

## Abstract

Phlorofucofuroeckol A (PFF-A), one of the phlorotannins found in brown algae, has been reported to exert anti-cancer property. However, the molecular mechanism for the anti-cancer effect of PFF-A has not been known. Activating transcription factor 3 (ATF3) has been reported to be associated with apoptosis in colorectal cancer. The present study was performed to investigate the molecular mechanism by which PFF-A stimulates ATF3 expression and apoptosis in human colorectal cancer cells. PFF-A decreased cell viability through apoptosis of human colorectal cancer cells. PFF-A increased ATF3 expression through regulating transcriptional activity. The responsible *cis*-element for ATF3 transcriptional activation by PFF-A was cAMP response element binding protein (CREB), located between positions −147 and −85 of the ATF3 promoter. Inhibition of p38, c-Jun *N*-terminal kinases (JNK), glycogen synthase kinase (GSK) 3β, and IκB kinase (IKK)-α blocked PFF-A-mediated ATF3 expression. ATF3 knockdown by ATF3 siRNA attenuated the cleavage of poly (ADP-ribose) polymerase (PARP) by PFF-A, while ATF3 overexpression increased PFF-A-mediated cleaved PARP. These results suggest that PFF-A may exert anti-cancer property through inducing apoptosis via the ATF3-mediated pathway in human colorectal cancer cells.

## 1. Introduction

In metazoans, apoptosis has been used as a mechanism for regulating tissue homeostasis via eliminating redundant or potentially deleterious cells [1]. Induction of apoptosis has been arguably regarded as the most potent defense system against cancer. Most anti-cancer agents suppress cancer cell growth by inducing apoptosis [2,3]. Animal studies have suggested that some chemopreventive agents such as epigallocatechin gallate [4] sulindac [5], curcumin [5], quercetin [6], and capsaicin [7] can induce apoptosis in cancer cells *in vivo*. In addition, an association between a clinical response and apoptosis in cancer cells has been demonstrated in human chemoprevention trials [8,9]. Thus, induction of apoptosis in cancer cells has been regarded as an effective method of cancer prevention.

Activating transcription factor 3 (ATF3), a member of the ATF/cAMP response element binding protein (CREB) family of bZIP transcription factors, is a stress-responsive gene product [10] and has been reported to contribute to protective property against oxidative damage in astrocytes [11]. In contrast to astrocytes, ATF3 can function as a pro-apoptotic protein and mediate apoptosis by anti-cancer agents including nonsteroidal anti-inflammatory drugs (NSAID) [12], conjugated linoleic acid [13,14], LY294002 [15], curcumin [16], and 3,3’-diindolylmethane [17] in cancer cells. In terms of the importance of apoptosis to cancer prevention, ATF3 can be thought of as an important molecular target in the induction of apoptosis.

Phlorofucofuroeckol A (PFF-A), one of the phlorotannins found in brown algae, has various pharmacological properties such as antioxidant [18,19,20], anti-inflammatory [20,21], anti-plasmin [22], anti-acetylcholinesterase [23], and anti-allergic activities [24]. Although PFF-A has been reported to exert an anti-cancer effect [25], the more detailed mechanisms by which PFF-A plays a role in cancer prevention are still unknown. According to the literature on the anti-cancer activity of PFF-A [25], we evaluated the effect of PFF-A on the inhibition of cell viability and apoptosis in human colorectal cancer cells, and found that PFF-A decreased the cell viability and induced apoptosis. In addition, in the search for molecular targets, we found that PFF-A upregulated ATF3 expression. Thus, in this study, we investigated the molecular mechanism of PFF-A-mediated ATF3 expression and apoptosis in human colorectal cancer cells. The findings showed that PFF-A may be a potential candidate for human colorectal cancer prevention.

## 2. Results

### 2.1. Effect of PFF-A on the Viability of Human Colorectal Cancer Cells

**Figure 1:**
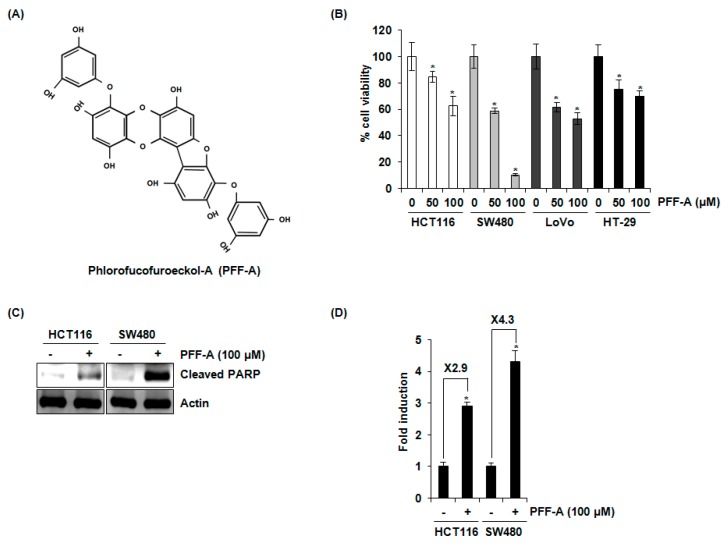
Molecular structure and the effect of phlorofucofuroeckol A (PFF-A) on cell viability and apoptosis in human colorectal cancer cells. (**A**) Molecular structure of PFF-A was shown; (**B**) human colorectal cancer cell lines such as HCT116, SW480, LoVo, or HT-29 cells were treated with PFF-A for 24 h. Cell viability was measured using 3-(4,5-dimethylthiazol-2-yl)-2,5-diphenyltetrazolium bromide (MTT) assay system and expressed as % cell viability. * *p* < 0.05 compared to cells without PFF-A; (**C**) HCT116 or SW480 cells were treated with 100 μM of PFF-A for 24 h. Cell lysates were subjected to sodium dodecyl sulfate polyacrylamide gel electrophoresis (SDS-PAGE) and Western blot was performed using antibodies against cleaved poly (ADP-ribose) polymerase (PARP) and actin; (**D**) for the ELISA analysis of cell death, the cytosol fraction was extracted from PFF-A-treated cells, and the cell death was measured using the Cell Death Detection ELISA^PLUS^ Kit, and expressed as Fold induction of absorbance (A_405_–A_490_). * *p* < 0.05 compared to cells without PFF-A treatment.

Phlorofucofuroeckol A (PFF-A) (Figure 1A), which is 4,9-bis(3,5-dihydroxyphenoxy)benzofuro(3,2-a)oxanthrene-1,3,6,10,12-pentol), has been known to have a molecular formula of C_30_H_18_O_14_ and a molecular weight of 602.46 Da [26]. To evaluate the effect of PFF-A on the viability of human colorectal cancer cells, the cells were treated with 0, 50, and 100 μM of PFF-A for 24 h and then cell viability was measured by 3-(4,5-dimethylthiazol-2-yl)-2,5-diphenyltetrazolium bromide (MTT) assay. As shown in Figure 1B, PFF-A attenuated the cell viability by 16% and 42% at 50 μM, and 38% and 90% at 100 μM in HCT116 and SW480 cells, respectively. Furthermore, the viability of LoVo and HT-29 cells was decreased by 39% and 25% at 50 μM of PFF-A, and 47% and 31% at 100 μM of PFF-A, respectively. To determine the association of apoptosis with the reduction of cell viability by PFF-A, cleaved PARP as the bio-marker of apoptosis was detected by Western blot after PFF-A treatment. As shown in Figure 1C, PFF-A increased the cleavage of PARP in HCT116 and SW480 cell. To confirm the effect of PFF-A on the induction of apoptosis, we performed a cell death assay using a Cell Death Detection ELISA^PLUS^ Photometric Enzyme Immunoassay Kit for the quantitative *in vitro* determination of cytoplasmic histone-associated DNA fragments. As shown in Figure 1D, PFF-A treatment increased the cell death by 2.9- and 4.3-fold in HCT116 and SW480 cells, respectively.

### 2.2. Effect of PFF-A on ATF3 Expression in Human Colorectal Cancer Cells

**Figure 2:**
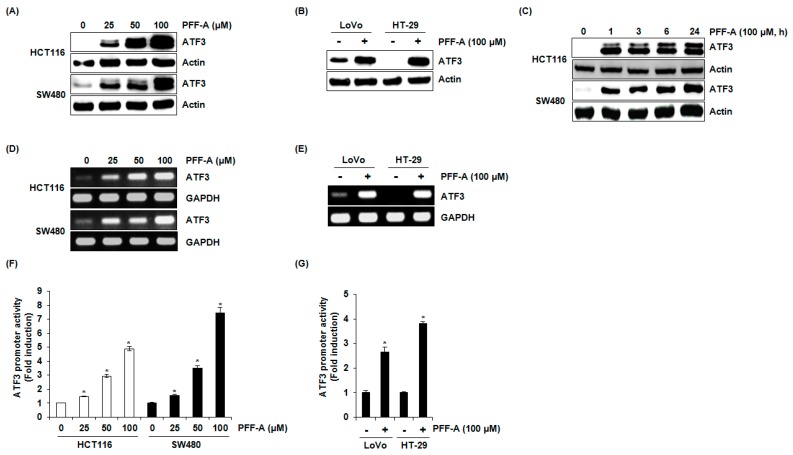
The effect of phlorofucofuroeckol A (PFF-A) on activating transcription factor 3 (ATF3) expression. (**A**,**B**) HCT116, SW480, LoVo, or HT-29 cells were treated with PFF-A for 24 h; (**C**) HCT116 and SW480 cells were treated with 100 μM of PFF-A for the indicated times. Cell lysates were subjected to SDS-PAGE and Western blot was performed using antibodies against ATF3 and actin; (**D**,**E**) HCT116, SW480, LoVo, or HT-29 cells were treated with PFF-A for 24 h, and then total RNA was isolated and RT-PCR was performed against ATF3 and glyceraldehyde-3-phosphate dehydrogenase (GAPDH); (**F**,**G**) the pATF3-1420/+34 construct (1 μg) was co-transfected with pRL-null vector (0.1 μg) into HCT116, SW480, LoVo, or HT-29 cells. The cells were treated with PFF-A for 24 h and then the luciferase activity was measured. * *p* < 0.05 compared to cells without PFF-A.

As shown in Figure 2A,B, PFF-A dose-dependently increased ATF3 protein level in HCT116 and SW480 cells. In addition, PFF-A-mediated increase of ATF3 protein was observed in LoVo and HT-29 cells. In a time-course experiment (Figure 2C), ATF3 overexpression started to increase 1 h after PFF-A treatment in HCT116 and SW480 cells. To evaluate whether the increase of ATF3 protein by PFF-A contributed to transcriptional regulation, the level of ATF3 mRNA was measured. Similarly to the effect of PFF-A on the level of ATF3 protein, PFF-A amplified the expression of ATF3 mRNA in HCT116, SW480, LoVo, and HT-29 cells (Figure 2D,E). To confirm the effect of PFF-A on ATF3 transcriptional activation, the change of ATF3 promoter activity by PFF-A was tested. As shown in Figure 2F,G, ATF3 promoter activation by PFF-A treatment was observed in HCT116, SW480, LoVo, and HT-29 cells.

### 2.3. Identification of Cis-Acting Element Responsible for PFF-A-Induced ATF3 Activation

**Figure 3:**
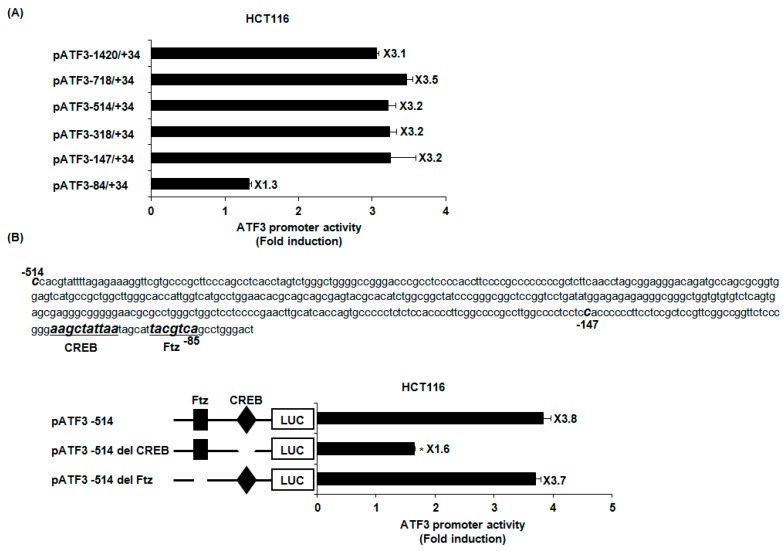
Identification of activating transcription factor 3 (ATF3) promoter sites responsible for phlorofucofuroeckol A (PFF-A)-induced ATF3 transcriptional activation. (**A**,**B**) Each indicated construct of the ATF3 promoter (1 μg) was co-transfected with 0.1 μg of pRL-null vector into HCT116, and then cells were treated with 100 μM of PFF-A. Luciferase activity was measured. * *p* < 0.05 compared to cells transfected with luciferase construct containing −1420 to +34 of human ATF3 promoter region or pATF3-514 wild-type.

To search the specific ATF3 promoter region for ATF3 activation by PFF-A, ATF3 promoter activity was measured using different sizes of ATF3 promoter luciferase constructs (pATF3-1420/+34, pATF3-718/+34, pATF3-514/+34, pATF3-318/+34, pATF3-147/+34, and pATF3-84/+34). As shown in Figure 3A, PFF-A treatment resulted in an increase of promoter activity. The fold induction was 3.1, 3.5, 3.2, 3.2, 3.2, and 1.3 in pATF3-1420/+34, pATF3-718/+34, pATF3-514/+34, pATF3-318/+34, pATF3-147/+34, and pATF3-84/+34, respectively. Because fold inductions of luciferase activities by PFF-A were lowest in cells transfected with pATF3-84/+34, the promoter region of ATF3 at −147/−85 may be responsible for PFF-A-induced ATF3 activation. The Fushi tarazu (Ftz) and CREB have been reported to be *cis*-acting elements in ATF3 promoter containing −147 to −85 [27]. To identify the role of each *cis*-acting element, each site-deleted ATF3 promoter construct was transfected into HCT116 cells and treated with 100 μM of PFF-A. As shown in Figure 3B, ATF3 promoter activity by PFF-A significantly decreased when the CREB site was deleted. However, the deletion of Ftz sites did not affect ATF3 promoter activity by PFF-A. These data indicate that CREB may be important for ATF3 promoter activation by PFF-A.

### 2.4. Upstream Kinases Associated with the Increase of ATF3 Expression by PFF-A

**Figure 4:**
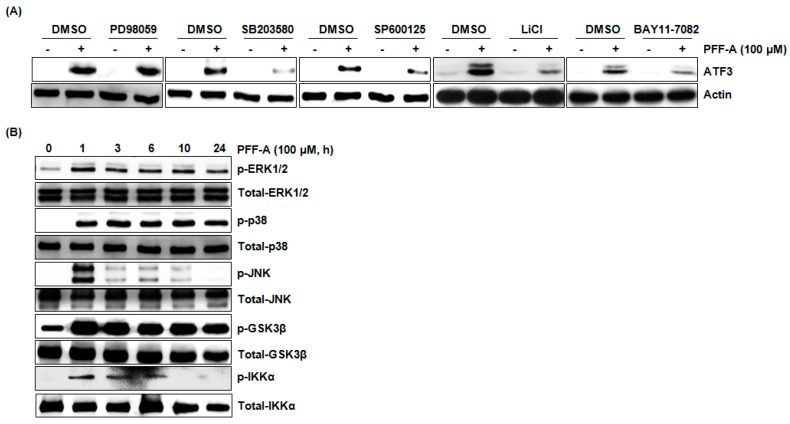
Identification of the upstream kinases involved in phlorofucofuroeckol A (PFF-A)-mediated activating transcription factor 3 (ATF3) expression. (**A**) HCT116 cells were pretreated with PD98059 (40 μM, ERK1/2 inhibitor), SB203580 (40 μM, p38 inhibitor), SP600125 (40 μM, JNK inhibitor), LiCl (20 mM, GSK3β inhibitor), or BAY11-7082 (10 μM, IKK-α inhibitor) for 2 h and then co-treated with 100 μM of PFF-A for 1 h. Cell lysates were subjected to SDS-PAGE and Western blot was performed using antibodies against ATF3 or actin; (**B**) HCT116 cells were treated with 100 μM of PFF-A for the indicated times. Cell lysates were subjected to sodium dodecyl sulfate polyacrylamide gel electrophoresis (SDS-PAGE) and Western blot was performed using antibodies against p-ERK1/2, total-ERK1/2, p-p38, total-p38, p-JNK, total-JNK, p-GSK3β, total-GSK3β, p-IKK-α, or total-IKK-α.

To elucidate the upstream kinases associated with ATF3 expression by PFF-A, each kinase inhibitor such as PD98059 (Extracellular signal-regulated kinases (ERK) 1/2 inhibitor), SB203580 (p38 inhibitor), SP600125 (JNK inhibitor), LiCl (GSK3β inhibitor), or BAY11-7082 (IKK-α inhibitor) was pretreated in HCT116 cells and then co-treated with PFFA. As shown in Figure 4A, inhibition of p38, JNK, GSK3β, and IKK-α attenuated the PFF-A-mediated ATF3 expression. However, inhibition of ERK1/2 did not affect ATF3 expression by PFF-A. These data indicate that activations of p38, JNK, GSK3β, and IKK-α may contribute to ATF3 expression. Thus, we investigated whether PFF-A activates these kinases and found that PFF-A induced the phosphorylation of p38, JNK, GSK3β, and IKK-α as an active modification of each kinase (Figure 4B).

### 2.5. ATF3 Mediates the Induction of Apoptosis by PFF-A

**Figure 5:**
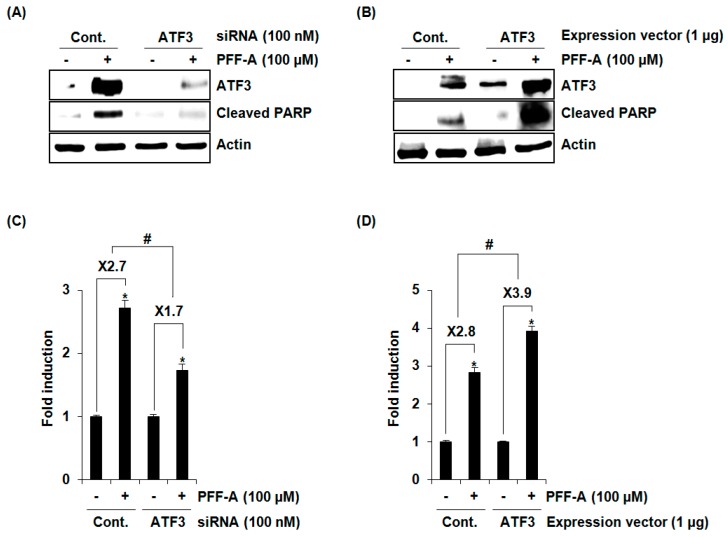
The effect of activating transcription factor 3 (ATF3) on phlorofucofuroeckol A (PFF-A)-mediated apoptosis. (**A**) ATF3 siRNA was transfected into HCT116 for 48 h and then 100 μM of PFF-A was treated for 24 h; (**B**) HCT116 cells was transfected with an empty or ATF3 expression vector for 24 h and then treated with 100 μM of PFF-A for 24 h. Cell lysates were subjected to SDS-PAGE and Western blot was performed using antibodies against ATF3, cleaved PARP, or actin. (**C**,**D**) For the ELISA analysis of cell death, the cytosol fraction was extracted from PFF-A-treated cells transfected with ATF3 siRNA or ATF3 overexpression vector, and the cell death was measured using the Cell Death Detection ELISA^PLUS^ Kit, and expressed as Fold induction of absorbance (A_405_–A_490_). * *p* < 0.05 compared to cells without PFF-A treatment. ^#^ *p* < 0.05 compared to PFF-A-treated cells transfected with control-siRNA or control-expression vector.

To evaluate whether overexpression of ATF3 by PFF-A affects apoptosis, Western blot against cleaved PARP was performed after ATF3 knockdown using ATF3 siRNA. As shown in Figure 5A, ATF3 knockdown attenuated PFF-A-mediated cleavage of PARP. To confirm the effect of ATF3 on apoptosis by PFF-A, ATF3 was overexpressed using an ATF3 expression vector and then cleaved PARP was measured using Western blot after PFF-A treatment. As a result (Figure 5B), PFF-A-mediated cleavage of PARP was aggravated in the cells transfected with an ATF3 expression vector compared with the cells transfected with a control expression vector. In addition, we confirmed the contribution of ATF3 to PFF-A-mediated apoptosis using a Cell Death Detection ELISA^PLUS^ Photometric Enzyme Immunoassay Kit, and found that PFF-A-induced cell death was decreased by ATF3 knockdown by ATF3 siRNA and increased by ATF3 overexpression (Figure 5C,D). These findings indicate that ATF3 may be a key factor for apoptosis by PFF-A.

## 3. Discussion

Induction of apoptosis has been long regarded as one of the most effective strategies in cancer prevention and therapy. Thus, many secondary metabolites from vegetables, herbal plants, and microorganisms have been studied for their proapoptotic properties. Phlorofucofuroeckol A (PFF-A), used in this study, is one of the phlorotannins found in brown algae. Although PFF-A has been reported to have various pharmacological properties including anti-cancer activity, the molecular mechanism of PFF-A remains unknown. In this study, we have elucidated the specific mechanism focused on ATF3 of PFF-A for apoptosis because ATF3 has been reported as a key factor for the induction of apoptosis by many phytochemicals in human colorectal cancer.

In cancer development, ATF3 has been reported to exhibit dual functions such as tumor suppressor or tumor progression, depending on cell context. In human colorectal tumors, ATF3 expression is repressed compared to normal adjacent tissue [28], and ATF3 overexpression can induce apoptosis in colorectal cancer cells [15]. In addition, ATF3 has been reported to enhance p53 activation [29,30], inhibit Ras-mediated tumorigenesis [31], and downregulate cyclin D1 and matrix metallopeptidase (MMP)-2 expression [32]. Furthermore, the therapeutic activity of heat shock protein 90 inhibition [33] and the antimetastatic activity of *N*-myc downstream regulated 1 (NDRG1) and Kallmann syndrome 1 (KAl1) can be mediated by ATF3 [34]. On the other hand, ATF3 can induce DNA synthesis and expression of cyclin D1 in hepatocytes [35] and is involved in cell proliferation as a target gene of c-myc [36]. In breast cancer, ATF3 enhances cancer’s cell-initiating features [37] and is associated with activation of the canonical Wnt/β-catenin pathway [38].

We showed that PFF-A dose-dependently decreased the cell viability in human colorectal cancer cells such as HCT116, SW480, LoVo, and HT-29 cells. Furthermore, the cleavage level of PARP as the biomarker of apoptosis and apoptosis-dependent cell death were increased by PFF-A treatment, indicating that PFF-A may induce apoptosis-mediated reduction of cell viability in human colorectal cancer cells. In addition, we found that PFF-A activates ATF3 expression via upregulating transcriptional activity, and knockdown of ATF3 by siRNA or ATF3 overexpression decreases or increases the cleavage of PARP by PFF-A, respectively. These findings suggest that ATF3 may be an important molecular target for PFF-A-mediated apoptosis. Interestingly, we found that SW480 cells were much more sensitive to the decrease of cell viability and the activation of ATF3 promoter by PFF-A compared to the other three cells at 100 μM, which also indicates that the inhibition of the cell viability and induction of apoptosis may be dependent on ATF3. However, we do not exclude other signaling associated with the inhibition of the cell viability and induction of apoptosis.

The ATF3 promoter contains a variety of response elements [10] and the transcription of the ATF3 gene is regulated by several mechanisms involving other CREB/ATF transcription factors [12,17]. In fact, some dietary compounds such as tolfenamic acid, 3,3′-diindolylmethane, and EGCG have been reported to activate ATF3 transcription through ATF2, ATF4, or EGR-1-dependent mode, respectively [12,17,27]. Interestingly, unlikely tolfenamic acid, 3,3′-diindolylmethane, and EGCG, we found that the promoter region responsible for ATF3 transcription by PFF-A is localized between −147 and −85, implying that another transcriptional mechanism might be involved in transactivation of ATF3 gene by PFF-A. There is growing evidence that the *cis*-acting elements in ATF3 promoter containing −147 to −85 are Fushi tarazu (Ftz) and CREB [27]. In this study, we found that the deletion of the CREB binding site in the ATF3 promoter significantly attenuated the increase of ATF3 promoter activity by PFF-A, implying that CREB may be associated with PFF-A-mediated ATF3 promoter activation.

ATF3 expression has been reported to be dependent on the activation of various kinases including MAPK (ERK1/2, p38 and JNK) [12,39], GSK3β [13], and IKK-α [40]. In this study, we found that inhibition of p38 by SB203580, JNK by SP600125, GSK3β by LiCl, and IKK-α by BAY11-7082 attenuates PFF-A-induced ATF3 expression, which p38, JNK, GSK3β, and IKK-α may be important kinases for activating ATF3 expression by PFF-A. In the relationship between p38, JNK, GSK3β, and IKK-α, and CREB, p38 has been reported to mediate the activation of CREB [41]. GSK3β phosphorylates CREB at Ser129 and subsequently induces CREB transcriptional activity [42]. However, there is no report that JNK and IKK-α directly or indirectly affect CREB activation. Thus, further study is required to explain in detail how JNK and IKK-α affect CREB activation. Although ERK1/2 has been regarded as an important regulator of ATF3 expression and PFF-A activated ERK1/2, ATF3 expression by PFF-A did not be changed by the inhibition of ERK1/2 by PD98059. However, we do not exclude the possibility that PFF-A may overcome the effect of the ERK1/2 inhibitor (PD98059). In addition, further study is required to explain in detail how ATF3 interacts with p38, JNK, GSK3β, or IKK-α in response to PFF-A. Interestingly, we found that the phosphorylation of JNK and IKK-α occurred over a short time, while the phosphorylation of the other three kinases was induced for a longer time. Although we do not understand why PFF-A time-differently phosphorylates these kinases, the important point in this study may be that PFF-A increases ATF3 expression in a p38-, JNK-, GSK3β-, or IKKα-dependent manner.

## 4. Materials and Methods

### 4.1. Chemical Reagents

Dulbecco’s Modified Eagle medium (DMEM)/F-12 1:1 Modified medium (DMEM/F-12) was purchased from Lonza (Walkersville, MD, USA). Antibodies against ATF3, poly (ADP-ribose) polymerase (PARP), p-ERK1/2, total-ERK1/2, p-p38, total-p38, p-JNK, total-JNK, p-GSK3β, total-GSK3β, p-IKKα, total-IKKα, and β-actin were purchased from Cell Signaling (Beverly, MA, USA). PD98059 (ERK1/2 inhibitor), SB203580 (p38 inhibitor), SP600125 (JNK inhibitor), LiCl (GSK3β inhibitor), and BAY11-7082 (IKKα inhibitor) were purchased from Calbiochem (San Diego, CA, USA). ATF3 siRNA was purchased from Santa Cruz Biotechnology (Santa Cruz, CA, USA). ATF3 promoter constructs used in this study were kindly provided by Seong Ho Lee (University of Maryland, College Park, MD, USA). All chemicals were purchased from Fisher Scientific, unless otherwise specified.

### 4.2. Cell Culture and Treatment

Human colorectal cancer cell lines (HCT116, SW480, LoVo and HT-29) were purchased from the Korean Cell Line Bank (Seoul, Korea) and grown in DMEM/F-12 supplemented with 10% fatal bovine serum (FBS), 100 U/mL penicillin, and 100 μg/mL streptomycin. The cells were maintained at 37 °C under a humidified atmosphere of 5% CO_2_. Phlorofucofuroeckol A (PFF-A, purity >99%) was isolated from the edible brown alga *Eisenia bicyclis* [43], dissolved in dimethyl sulfoxide (DMSO) and added to cells. DMSO was used as a vehicle and the final DMSO concentration did not exceed 0.1% (*v*/*v*).

### 4.3. Cell Viability Assay

Cell viability was measured using an MTT assay system. Briefly, cells were plated onto 96-well plates and grown overnight. The cells were treated with 0, 50, and 100 μM of PFF-A for 24 h. Then, the cells were incubated with 50 μL of MTT solution (1 mg/mL) for an additional 2 h. The resulting crystals were dissolved in DMSO. The formation of formazan was measured by reading absorbance at a wavelength of 570 nm.

### 4.4. Cell Death Assay

Cell death was measured using a Cell Death Detection ELISA^PLUS^ Kit (Roche Diagnostics, Indianapolis, IN, USA) according to the manufacturer’s instruction. Briefly, cells were plated in a six-well plate. After 24 h, cells were treated with PFF-A for 24 h. After the treatment, the cytosol was prepared using a Nuclear Extract Kit (Active Motif, Carlsbad, CA, USA). Equal amounts of cytosolic extracts, immunoreagent containing anti-histone-biotin, and anti-DNA-POD were added to a microplate well and incubated for 2 h under shaking. After washing, the ABTS solution was added to each well for 20 min and then the ABTS stop solution was added. The absorbance was recorded at 405 nm and 490 nm in an enzyme-linked immunosorbent assay plate reader.

### 4.5. Reverse Transcriptase-Polymerase Chain Reaction (RT-PCR)

Total RNA was prepared using a RNeasy Mini Kit (Qiagen, Valencia, CA, USA) and 1 μg of total RNA was reverse-transcribed using a Verso cDNA Kit (Thermo Scientific, Pittsburgh, PA, USA) according to the manufacturer’s protocol for cDNA synthesis. PCR was performed using a PCR Master Mix Kit (Promega, Madison, WI, USA) with human primers for ATF3 and glyceraldehyde-3-phosphate dehydrogenase (GAPDH) as followed: human ATF3: 5′-gtttgaggattttgctaacctgac-3′, and reverse 5′-agctgcaatcttatttctttctcgt-3′; human GAPDH: forward 5′-acccagaagactgtggatgg-3′ and reverse 5′-ttctagacggcaggtcaggt-3′.

### 4.6. Transient Transfections

Transient transfections were performed using the PolyJet DNA transfection reagent (SignaGen Laboratories, Ijamsville, MD, USA) according to the manufacturer’s instruction. Cells were plated in 12-well plates at a concentration of 2 × 10^5^ cells/well. After growth overnight, plasmid mixtures containing 1 μg of ATF3 promoter linked to luciferase and 0.1 μg of pRL-null vector were transfected for 24 h. The cells were then harvested in 1 × luciferase lysis buffer, and luciferase activity was normalized to the pRL-null luciferase activity using a dual-luciferase assay kit (Promega, Madison, WI, USA).

### 4.7. Transfection of Small Interference RNA (siRNA)

HCT116 cells were plated in six-well plates and incubated overnight. HCT116 cells were transfected with control siRNA and ATF3 siRNA for 48 h at a concentration of 100 nM using a TransIT-TKO transfection reagent (Mirus, Madison, WI, USA) according to the manufacturer’s instruction.

### 4.8. Expression Vector

ATF3 expression vector was provided by Addgene (Cambridge, MA, USA). Transient transfection of the vector was performed using the PolyJet DNA transfection reagent (SignaGen) according to the manufacturer’s instruction.

### 4.9. SDS-PAGE and Western Blot

The cells were washed with 1× phosphate-buffered saline (PBS), and lysed in radioimmunoprecipitation assay (RIPA) buffer (Boston Bio Products, Ashland, MA, USA) supplemented with protease inhibitor cocktail (Sigma-Aldrich, St. Louis, MO, USA) and phosphatase inhibitor cocktail (Sigma-Aldrich), and centrifuged at 15,000× *g* for 10 min at 4 °C. After determining the protein concentration by a bicinchoninic acid (BCA) protein assay (Pierce, Rockford, IL, USA), the proteins were separated via SDS-PAGE and transferred to a PVDF membrane (Bio-Rad Laboratories, Inc., Hercules, CA, USA). The membranes were blocked for non-specific binding with 5% non-fat dry milk in Tris-buffered saline containing 0.05% Tween 20 (TBS-T) for 1 h at room temperature and then incubated with specific primary antibodies in 5% non-fat dry milk at 4 °C overnight. After three washes with TBS-T, the blots were incubated with horseradish peroxidase (HRP)-conjugated immunoglobulin G (IgG) for 1 h at room temperature and the chemiluminescence was detected using an ECL Western blotting substrate (Amersham Biosciences, Piscataway, NJ, USA) and visualized on Polaroid film.

### 4.10. Statistical Analysis

All the data are shown as mean ± SEM (standard error of mean). Statistical analysis was performed with one-way analysis of variance (ANOVA) followed by Dunnett’s test. Differences with * *p* < 0.05 were considered statistically significant.

## 5. Conclusions

The current data demonstrate that PFF-A increases ATF3 expression through transcriptional regulation, which might be associated with the induction of apoptosis in human colorectal cancer cells. In addition, the current study can provide information for the molecular target of PFF-A’s anti-cancer activity.
